# Visual Inspection with Acetic Acid Positivity in Screening and Early Detection of Cervical Dysplasia in Africa, 2023: A Meta-Analysis

**DOI:** 10.4314/ejhs.v34i1.2

**Published:** 2024-01

**Authors:** Yohannes Fikadu Geda, Yirgalem Yosef Lamiso, Tamirat Melis Berhe, Seid Jemal Mohammed, Samuel Ejeta chibsa, Daniel Adane Endalew, Kenzudin Assfa Mossa, Seblework Abeje, Mustefa Adem Hussen, Molalign Mesele Gesesse

**Affiliations:** 1 Wolkite University, Ethiopia; 2 Mattu University, Ethiopia; 3 Injibara University, Ethiopia; 4 Wolaita Sodo University, Ethiopia

**Keywords:** Africa, cervical cancer, cervical dysplasia, Visual inspection with acetic acid

## Abstract

**Background:**

Visual Inspection with Acetic acid (VIA) is the best feasible method of screening and early detecting for cervical dysplasia for resource limited settings like Africa. There is no study that can represent Africa on VIA positivity. Therefore, this metaanalysis was planned to verify the best available articles to pool the visual inspection with acetic acid positivity in screening and early detection of cervical dysplasia in Africa.

**Methods:**

The Cochrane Library, Web of Science, PubMed, Scopus, free Google database search engines, Google Scholar, and Science Direct databases were used to conduct a true search of this research article. STATA version 14.0 was used to do the metaanalysis. This meta-analysis was registered in PROSPERO database under the identity pf CRD42023392197.

**Result:**

This meta-analysis analyzed data from 21,066 women who had VIA examination to estimate the pooled VIA positivity in Africa. The overall pooled effect estimate of VIA positivity in Africa was 11.93 (95%CI: 11.48–12.37). Age <16 year during first intercourse 2.58(95%CI: 1.53–3.62), lifetime sexual partner ≥2 3.92(95%CI: 2.05–5.78) and HIV positivity 2.92(95%CI: 1.72–4.12) were the significant variables which influence VIA positivity.

**Conclusion:**

Overall pooled effect estimate of VIA positivity in Africa was high compared to other continents. The main factors that affect VIA positivity are age at first sexual contact being under 16 years old, the number of lifetime sexual partners being at least two, and HIV positivity. Therefore, the WHO's goal of creating Africa free of cervical cancer is still one that requires significant effort.

## Introduction

Cervical cancer remains a major public health problem throughout the world (1). Cancer of the cervix is the fourth most commonly diagnosed cancer and the fourth most common cause of cancer mortality in women. The World Report on Cancer Incidence, Mortality and Prevalence estimates that there were about 570,000 cases and 311,000 deaths of cervical cancer worldwide in 2018 (2). Globally, cervical cancer is expected to increase to nearly 700,000 cases and 400,000 deaths by 2030 without any intervention, representing a 21% and 27% increase in the number of cases and deaths, respectively (3).

In Sub-Saharan Africa, approximately 19.59% of women aged 15 years and older cases, and 24.55% of deaths per year occur related to cervical cancer. In Sub-Saharan Africa, the incidence rates of cervical cancer in some countries are the highest in the world. Swaziland and Malawi have the highest incidence in Africa, with age-standardized incidence rates (ASR) of 75.3 and 72.9 per 100 000 women, respectively (4).

Human papillomavirus (HPV) is the necessary cause of cervical cancer. Persistent infection with high-risk types of HPV activates the progression of a normal cell into precancerous lesions leading to cervical cancer. HPV types 16 and 18 account for about 70% of cervical cancer cases globally (5). Socio-demographic factors, socioeconomic factors, sexual and reproductive health-related factors and medical or surgical comorbidities factors play different roles in the occurrence of precancerous cervical lesions and cervical cancer (6-8).

Convincing evidence confirmed that HPV vaccination programs for the most common high-risk HPV would prevent about 87% of cases of cervical cancer worldwide. Since the approval of HPV vaccination in 2006, about 80 countries and territories have implemented national HPV vaccination programmes, covering over 100 million women. In 2018, the World Health Organization (WHO) released a global call for the elimination of cervical cancer as a public health issue by this century. Today, more than ever, effective planning for the fight against cervical cancer requires a precise estimate of this disease (3).

Globally, there are different strategies to control the increase in the magnitude of cervical cancer. Screening for precancerous cervical lesions is one of these strategies. There are different methods of cervical cancer screening including HPV DNA test, Papanicolaou smear, and visual inspection with acetic acid (VIA) methods.

Screening with inspection with acetic acid in resource-limited settings is a commonly preferred method compared to HPV DNA test and cytology or Pap smear (9). WHO recommends VIA as the primary approach for cervical screening in resource-constrained settings. In principle, this screening method is a less complex technique and can be performed by trained healthcare providers with different backgrounds such as doctors, midwives and nurses. In sub-Saharan Africa, the uptake of cervical cancer screening was 12.87% (10).

Even though there were some amounts of primary articles conducted on visual inspection with acetic acid positivity in screening and early detection of cervical dysplasia in a resource-limited setting, and also there is no study that represent as reference in Africa. Therefore, this meta-analysis was planned to verify the best available articles to pool the visual inspection with acetic acid positivity in screening and early detection of cervical dysplasia in Africa.

## Methods

Study design and setting: The authors have assessed the PROSPERO database (https://www.crd.york.ac.uk/PROSPERO/) for all published or ongoing researches available related to the title to skip any further duplication. Accordingly, the result showed that there were no ongoing or published articles on this title.

Therefore, this meta-analysis was registered in the PROSPERO data base with an identification number of CRD42023392197 on 27/01/2023. The meta-analysis was conducted to verify the pooled VIA positivity in Africa. Scientific consistency was formulated by using PRISMA checklist.

**Information source**: A systematic and genuine search of the research articles was done via the following listed databases: PubMed, Scopus, Cochrane Library, the Web of Science, free Google databases search engines, Google Scholar, and Science Direct search engines. We used the keywords ((((((“cervical neoplasms” [Me SH Terms] OR (“uterine”[All Fields] AND “cervical”[All Fields] AND “neoplasms” [All Fields]) OR “cervical neoplasms”[All Fields] OR (“cervical”[All Fields] AND “cancer”[All Fields]) OR “cervical cancer” [All Fields]) AND (“diagnosis”[Subheading] OR “diagnosis” [All Fields] OR “screening”[All Fields] OR “early detection of cancer”[MeSH Terms] OR (“early”[All Fields] AND “detection” [All Fields] AND “cancer”[All Fields]) OR “early detection of cancer”[All Fields])) AND Visual Inspection with Acetic acid positivity [All Fields]) OR visual inspection with acetic acid positivity [All Fields]) OR (“statistics and numerical data”[Subheading] OR (“statistics”[All Fields] AND “numerical”[All Fields] AND “data”[All Fields]) OR “statistics and numerical data” [All Fields])) (“all African countries interchangeably” [MeSH Terms] OR “all African countries interchangeably” [All Fields]).

The search was performed using the following key search terms: “AND” and “OR” boolean operators individually and in combination with each other. In addition, reference lists for all included studies were consulted to identify any other studies that could have been missed by the search strategy. The search for all the research was made from October 10 to December 20, 2022 without limiting the publication dates of the literature.

**Inclusion criteria**: Papers published in national and international journals, as well as unpublished papers from institutional repositories conducted in Africa with results on VIA's positivity, were included in this study. Published and unpublished papers were sought and examined for inclusion in the final analysis. This study included available observational cross-sectional study models that report VIA positivity as well as case-control studies with the determinants of VIA positivity. All researches that were published, master's thesis found in institutional repositories, and PhD dissertation accessed from the repositories till the final date of data analysis and submission of this manuscript to this journal were included in accordance with these criteria.

**Figure 1:**
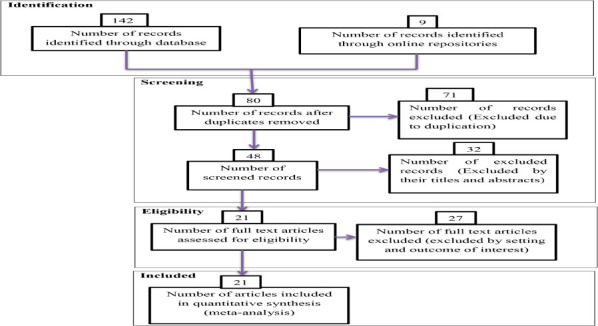
PRISMA flow diagrams of included studies in the VIA Positivity in screening and early detection of cervical dysplasia in Africa: A meta-analysis, 2023

Early in our research, 151 studies were identified, 71 of which were skipped due to duplication, and the remaining 80 studies were identified for eligibility. From 80 studies, 32 were excluded by highlight review on their abstracts, 48 studies assessed for full text from these 27 studies were excluded because of being not relevant to the current review, and the remaining 21 studies were included in the final meta-analysis (Figure 1).

**Exclusion criteria**: Studies that did not have proven methods were not included in this analysis. Articles that did not contain complete information important for analysis and case reports were excluded from this study. Duplicative study results and inconsistent measurements of outcome variables were excluded from the final analysis (Figure 1). Researches written in languages other than English were not included.

**Quality assessment and data extraction**: The baseline quality of the research articles included was assessed using the Newcastle-Ottawa scale (NOS). NOs were designed to assess the quality of observational research articles in meta-analyses (Table 1). Data of this study were extracted by the two authors (YFG and YYL) using a standardized data extraction checklist on excel sheet.

**Table 1:** Quality assessment of included studies on VIA positivity in screening and early detection of cervical dysplasia in Africa: A meta-analysis, 2023

Studies	Quality assessment criteria			
	Selection	Comparability	Outcome	Overall quality
Abdel-Hady et.al^11^	****	*	**	7
Albert et.al^12^	****	**	**	8
Awoke et.al^13^	****	*	**	7
Biaye et.al^14^	****	**	**	8
Dartell et.al(-)^15^	****	**	**	9
Dartell et.al(+)^15^	****	**	**	8
Deksissa et.al^16^	****	**	**	8
Desire et.al^17^	****	**	**	8
Fentie et.al^18^	****	*	**	7
Gad et.al^19^	****	*	**	7
Hend et.al^20^	****	**	**	8
Howieda et.al^21^	****	*	**	7
Huchko et.al^22^	****	*	**	7
Ibrahim et.al^23^	*****	**	**	9
Jeronimo et.al^24^	****	*	**	7
Mbamara et.al^25^	****	**	**	8
Namale et.al^26^	****	*	**	7
Olusegun et.al^27^	****	*	**	7
Tesfaye et.al^28^	***	*	**	6
Thérèse et.al^29^	***	*	**	6
Zekariase et.al^30^	****	**	**	8

The Newcastle Ottawa Scale (NOS) was used to assess the quality of included

** Two points, *** Three points; and **** four point

This meta-analysis uses the PRISMA flowchart to differentiate and select items of significance to the analysis. PRISMA is a minimal set of items for reporting in systematic reviews and meta-analyses that are based on evidence. Though it can serve as a foundation for publishing systematic reviews with objectives other than evaluating treatments, PRISMA is primarily concerned with the reporting of reviews evaluating the effects of interventions. Initially, duplicate types of studies were not included using the Endnote version X8.1 referencing tool. Articles were excluded by adding highlights by going through their titles and abstracts before evaluating the entire text. Full-text studies or research results were evaluated for other studies. Based on the aforementioned eligibility criteria, items were assessed for eligibility.

Data were extracted using the standardized data extraction tool in considering the name of the first author, publication year, country of study, author's affiliation, study type, population type and sample size (Table 2). All literacies were independently verified by the two authors (YFG and YYL). Where disagreements occurred, the articles were reviewed by one of the authors (SJM) and used as final mediation and admissibility decision.

**Table 2:** Descriptive summary of included articles on VIA Positivity in screening and early detection of cervical dysplasia in Africa: A meta-analysis, 2023

Authors	Year	Country	Affiliation of the authors	Study type	Population	Sample Size
**Abdel-Hady et.al** **11**	2006	Egypt	Mansoura University	CS	women aged 20-60	5000
**Albert et.al** **12**	2012	Nigeria	ABU Teaching Hospital	CS	postpartum mothers	359
**Awoke et.al** **13**	2019	Ethiopia	Bahir Dar University	CS	women aged≥30	428
**Biaye et.al** **14**	2019	Senegal	University Hospital Aristide Le Dantec	CS	RSW	899
**Dartell et.al(-)** **16**	2014	Tanzania	University of Copenhagen	CCS	HJV -ve women	3005
**Dartell et.al(+)** **16**	2014	Tanzania	University of Copenhagen	CCS	HIV +ve women	334
**Deksissa et.al** **17**	2015	Ethiopia	Columbia University	CS	women aged 25–15	334
**Desire et.al** **18**	2016	DRC	University of Lubumbashi	CS	women aged 22-67	229
**Fentie et.al** **19**	2020	Ethiopia	Addis Ababa University	CS	RSW	844
**Gad et.al** **31**	2019	Egypt	Al-Azhar University Hospital	CS	women aged 25-60	379
**Hend et.al** **20**	2016	Egypt	Zagazig University	CS	women aged 18-61	650
**Howieda et.al** **21**	unpu	Egypt	Assiut University	CS	RSW	450
**Huchko et.al** **32**	2015	Kenya	University of California	CS	HIV +ve women	1439
**Ibrahim et.al** **23**	2012	Sudan	University of Southern Denmark	CS	women aged 25-50	1250
**Jeronimo et.al** **33**	2014	Uganda	Program for Appropriate Technology in Health	CS	women aged 25-60	3146
**Mbamara et.al** **25**	2011	Nigeria	Nnamdi Azikiwe University Teaching Hospital Nnewi	CS	women aged 16-64	198
**Namale et.al** **34**	2021	Uganda	Uganda Virus Research Institute	CS	female sex workers	719
**Olusegun et.al** **27**	2016	Nigeria	Federal Teaching Hospital Ido-Ekiti	CS	women aged 20-70	220
**Tesfaye et.al** **28**	2022	Ethiopia	Save the Children	UCC	RSW	258
**Thérèse et.al** **29**	2022	Burkina Faso	Centre Muraz	CS	women aged 18-60	577
**Zekariase et.al** **30**	2015	Ethiopia	Mekelle Hospital	UCC	HJV +ve women	348

N. B: UCC=Un-matched case control, CCS=Comparative Cross-Sectional, CS=Cross-Sectional, Unpu=Un-published, DRC=Democratic Republic of Congo, −ve=Negative, +ve=Positive, RSW=Routinely Screened Women

**Data synthesis and analysis**: The analysis of this meta-analysis was conducted by STATA version 14.0. Quantitative reviews were conducted to determine the overall pooled VIA positivity in screening and early detection of cervical dysplasia in Africa. The degree of heterogeneity between the included studies was evaluated by determining the p-values of I^2^-test statistics. I^2^ test statistics scores of 0, 25, 50, and 75% were taken as no, low, moderate, and high degrees of heterogeneity, respectively. Due to the observed significant heterogeneity across studies, we used a random effect model to assess pooled estimate. Publication bias was checked by funnel plot. A p-value of less than 0.05 was used as cutoff point for statistical significance of publication bias. Egger test was done and verified that there were no small-study effects.

## Results

**Selection and characterization of included studies**: Twenty-one articles were included in this meta-analysis as summarized in Table 1. All studies were included in accordance with the eligibility criteria with the sample size ranging from 5000 in Egypt to 198 in Nigeria (Table 2).

Among the 54 countries of Africa, studies on VIA positivity were available only in 10 countries which fulfilled the inclusion criteria. The numbers of studies available were one study in Burkina Faso, one study in Democratic Republic of Congo, four studies in Egypt, five studies in Ethiopia, one study in Kenya, three studies in Nigeria, one study in Senegal, one study in Sudan, one study in Tanzania, and two studies in Uganda.

This meta-analysis analyzed data from 21,066 women who had VIA examination to estimate the pooled VIA positivity in Africa. All articles, published or unpublished, were included in this meta-analysis if they fulfilled the inclusion criteria (Table 2).

**Figure 2:**
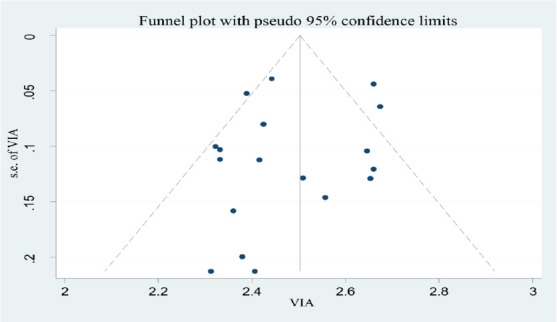
Funnel plot for studies on VIA Positivity in screening and early detection of cervical dysplasia in Africa: A meta-analysis, 2023

**Publication bias**: Bias among the included studies was checked by funnel plot at a 5% significant level. The funnel plot was symmetrical, and showed no statistical significance for the presence of publication bias for each study (Figure 2).

**Figure 3:**
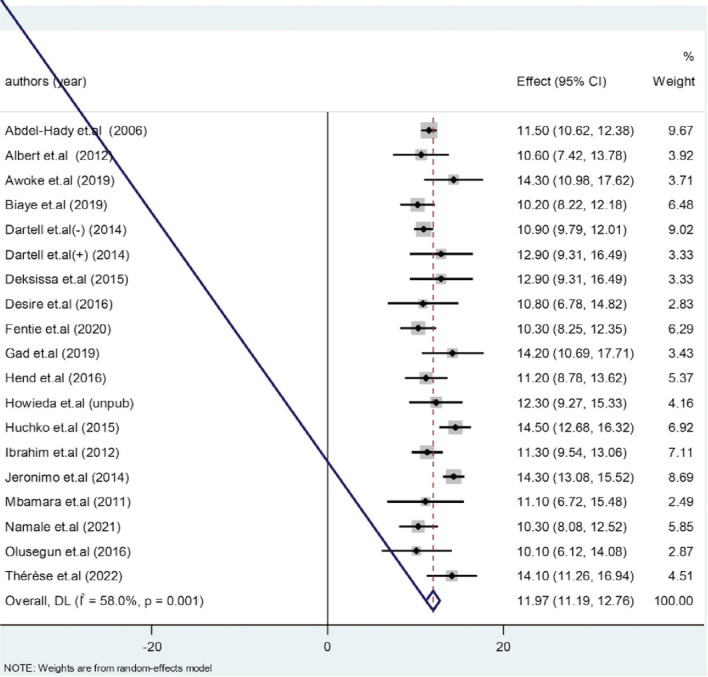
Forest plot for studies on VIA positive in screening and early detecten of cervical dysplasia in Africa. A meta-analysis 2023

**VIA positivity in Africa**: Eligible studies were included in the final meta-analysis. Due to observed moderate heterogeneity among the studies, random effect model was employed. The overall pooled effect estimate of VIA positivity in Africa was 11.93 with 95% confidence interval of 11.48 to 12.37 (Figure 3).

**Figure 4:**
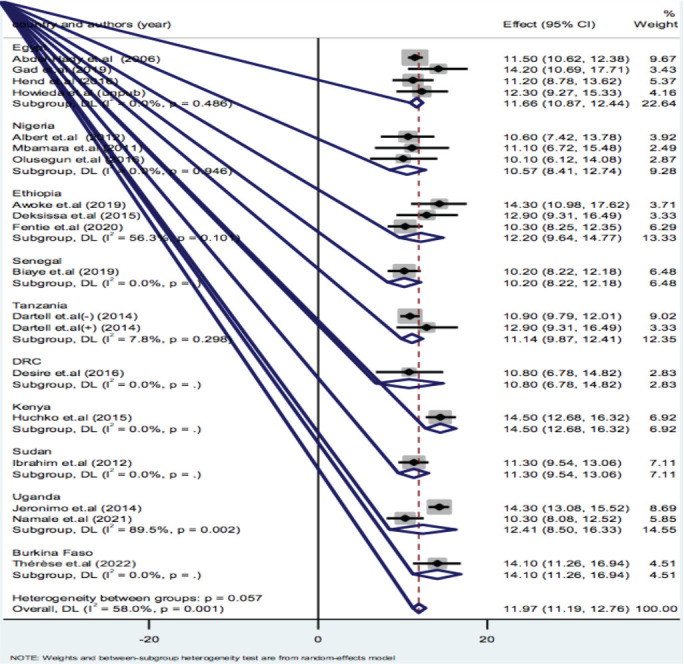
Subgroup analysis by Country on VIA Positivity in screening and early detection of cervical dysplasia in Africa: A meta-analysis, 2023

**Subgroup analysis of VIA positivity by country**: Subgroup analysis for pooled VIA positivity by country was done. Unfortunately, two or more studies were found on four countries. Accordingly, VIA positivity in Egypt was 11.66(95%CI: 10.87-12.44), in Nigeria was 10.57(95%CI: 8.41-12.74), in Ethiopia was 12.2 (95% CI: 9.64-37.08), and in Uganda was 12.41(95%CI: 8.50-16.33) (Figure 4).

**Figure 5:**
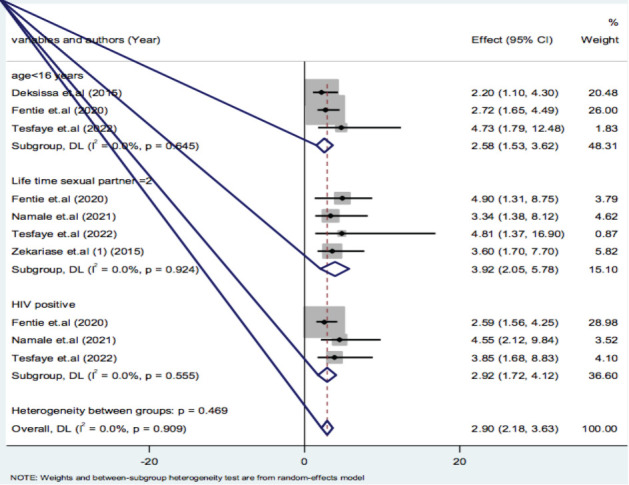
Subgroup analysis by variables on VIA Positivity in screening and early detection of cervical dysplasia in Africa: A meta-analysis, 2023

**Subgroup analysis of VIA positivity by variables**: Variables influencing VIA positivity were in parallel reviewed with the prevalence. Age less than 16 year during first intercourse with pooled effect estimate of 2.58(95%CI: 1.53-3.62), lifetime sexual partner greater than or equal to two with pooled effect estimate of 3.92(95%CI: 2.05-5.78) and HIV positivity with pooled effect estimate of 2.92(95%CI: 1.72-4.12) were the significant variables which influence VIA positivity (Figure 5).

## Discussion

According to WHO's recommendation, VIA is the primary approach for cervical screening in resource-constrained settings compared to HPV DNA test, cytology or Pap smear. This recommendation could result in improved cervical cancer prevention and more accurate identification of early-stage disease in this setting compared to existing cytology-based screening. In principle, this screening method is a less complex technique and can be performed by trained healthcare providers in different backgrounds with simple training. ACOG recommended that implementing alternative cervical cancer screening strategies like VIA in low-resource settings can provide consistent, accessible screening opportunities.

Studies conducted on all 54 countries of Africa, according to the United Nations, were extremely reviewed as it was stated on the search strategies. Available studies on VIA positivity in screening and early detection of cervical dysplasia in Africa were reviewed, which were limited to some list of countries.

All primary studies were included in this study irrespective of publication or study year. By default, the included studies were conducted from 2006 to 2022. According to Prospero database search, there was no meta-analysis conducted on VIA positivity in screening and early detection of cervical dysplasia in Africa.

In this study, a total of 21 eligible studies were accessed. Of them, 19 were cross-sectional studies and the remaining two were case-control studies.

This meta-analysis revealed that the overall pooled effect estimate of VIA positivity in Africa was 11.93 with 95% confidence interval of 11.48 to 12.37. Contrary to this, a study conducted in Asia reported that, out of 144 screened patients, 62(43.05%) were positive in visual inspection with acetic acid test (35). This big difference on those studies might be due to the study population of the Asian report, which were patients with symptom of cervical dysplasia.

This study analyzed that age less than 16 years during first intercourse was the predisposing factor for VIA positivity. In the same fashion, a study conducted in Europe reported similarly with this study (36). Since all previous and current studies have similar conclusions, this might verify that early first intercourse is an independent risk factor for cervical dysplasia.

This study showed that lifetime sexual partner greater than or equal to two was more likely to contract cervical dysplasia compared to one lifetime sexual partner. Similarly, a study suggested that having multiple sexual partners, with or without HPV infection, is a potential risk factor of cervical cancer (37). This might be due to the fact that the number sexual partners can increase the risk of contracting sexually transmitted infections by increasing exposure to HPV infection.

This study analyzed that HIV positivity were the significant variable which influence VIA positivity. Different studies also recommended higher frequency of VIA positivity in HIV-positive women. Which suggests HIV positivity predisposes to invasive cervical dysplasia on account of immunosuppression and co-existing HPV infection (38). This might be due to a decrease in immunity of HIV positive women or a higher exposure to HPV infection of women with HIV.

The overall pooled effect estimate of VIA positivity in Africa was high compared to other continents. Age less than 16 years during first intercourse, lifetime sexual partner greater than or equal to two, and HIV positivity were the significant variables which influence VIA positivity. Therefor:

The WHO's ambitious objective of achieving a cervical cancer-free future for Africa hinges on sustained efforts in implementing comprehensive screening and treatment programs. Central to this mission is the proactive utilization of the HPV vaccine as a pivotal tool in the eradication of cervical cancer, underscoring the critical importance of prevention. Addressing factors such as early age intercourse and premature marriages assumes paramount significance in the battle against cervical dysplasia in Africa. Governments must, therefore, take the initiative to launch mass campaigns across various platforms, including mass media, schools, and public gatherings, to raise awareness and curb early age intercourse exposure. Collaboration between the WHO and other sexual health organizations is essential to disseminate sexual health education within African communities through diverse channels. This educational outreach should advocate for the reduction of sexual partners to one and emphasize the imperative for health service providers and institutions to recommend cervical screening for all HIV-positive women. Such measures are pivotal in facilitating early treatment, thereby preventing the progression of additional severe diseases and contributing to the realization of the WHO's overarching vision for a cervical cancer-free Africa.
